# Supplementary material for: Exome-wide association study identifies KDELR3 mutations in extreme myopia
Source: Nat Commun. 2024 Aug 7;15:6703. doi: 10.1038/s41467-024-50580-x (PMC11306401; doi:10.1038/s41467-024-50580-x)
Supplement: Supplementary file 1 — Supplementary Information [file 41467_2024_50580_MOESM1_ESM.pdf]

Supplementary materials for

Exome-wide Association Study Identifies KDELR3 Mutations in Extreme Myopia

Jian Yuan, Youyuan Zhuang, Xiaoyu Liu, Yue Zhang, Kai Li, Zhen Ji Chen, Dandan Li, He Chen, Jiacheng Liang, Yinghao Yao, Xiangyi Yu, Ran Zhuo, Fei Zhao, Xiangtian Zhou, Myopia Associated Genetics and Intervention Consortium, Xiaoguang Yu, Jia Qu, Jianzhong Su

Correspondence to Jianzhong Su (sujz@wmu.edu.cn)

Supplementary figures

Supplementary Figure 1. Overview of mutations identified in known myopia genes.

Supplementary Figure 2. Exome-wide association study for single variants by EMMAX and MLMA-LOCO.

Supplementary Figure 3. Burden analysis for 449 EM cases and 9606 controls.

Supplementary Figure 4. Enrichment of rare variants in 10,587 gene sets derived from Human MSigDB Collections.

Supplementary Figure 5. Manhattan plot of MAGMA gene-based genetic association analysis on EM.

Supplementary Figure 6. Associations of single-cell of human eyes with EM.

Supplementary Figure 7. Gene expression of EM-associated fibroblasts.

Supplementary Figure 8. QQ plots for gene-level burden analysis by using FET.

Supplementary Figure 9. Overlap between cases with *KDELR3* PTVs and ones with diagnostic variants.

Supplementary Figure 10. Burden analysis of genes involved in retrograde vesicle-medicated

transport, Golgi to ER, when removed *KDELR1*, *KDELR2* or *KDELR3*.

Supplementary Figure 11. Spatiotemporal expression pattern of *kdelr3* in zebrafish.

Supplementary Figure 12. Morphology of *kdelr3*-deficient zebrafish morphants.

Supplementary Figure 13. RT-PCR confirmed expression change in *kdelr3*-MO injected zebrafish larvae.

Supplementary Figure 14. Sashimi plots illustrating target exon (exon 4) in *kdelr3-201* (GRCz11) in zebrafish eyeballs treated with *kdelr3* MOs (red sashimi plots) and controls MOs (blue sashimi plots).

Supplementary Figure 15. Quantification of eye axis length, the axis-to-body length ratio, lens diameter and lens-to-body length ratio for *kdelr3*-deficinet and rescue zebrafish.

Supplementary Figure 16. RNA-Seq data quality assessment.

Supplementary Figure 17. Heat map of Metascape-enriched clusters of upregulated targets in *KDELR3*-deficient transcriptome.

Supplementary Figure 18. qRT-PCR validation analysis.

Supplementary Figure 19. Western blot analysis.

Supplementary Figure 20. PheWAS Manhattan Plot.

Supplementary Figure 21. Distribution of spherical equivalent refraction of 449 EM.

Supplementary Figure 22. RNA-Seq data quality assessment.

Supplementary Figure 22. Initial sample quality control analysis.

Supplementary Figure 23. Distribution of the inbreeding coefficient.

Supplementary Figure 24. Principal component analysis with 1000 Genomes.

Supplementary Figure 25. PCA on EM cases and controls samples.

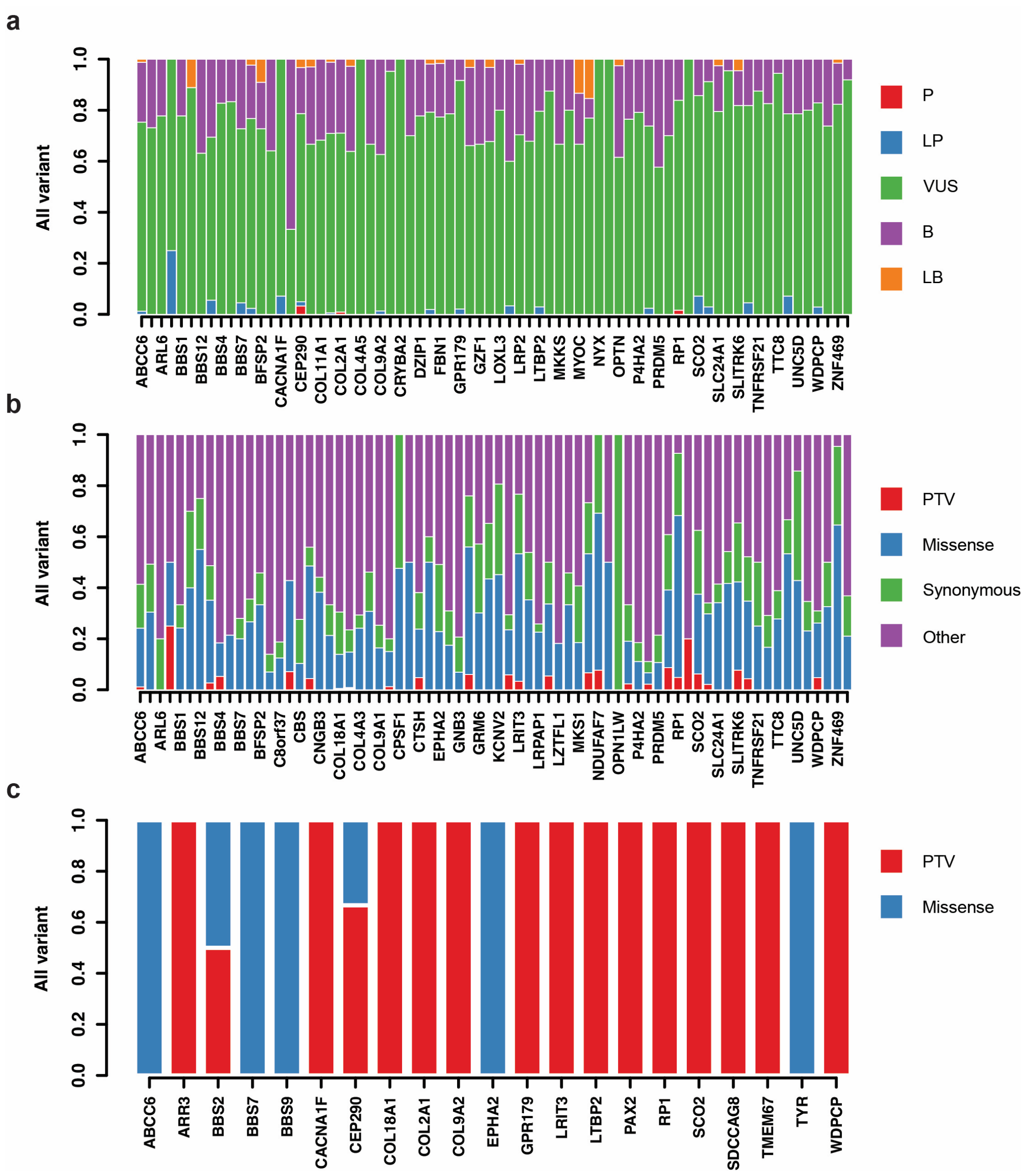

Supplementary Fig. 1. Overview of mutations identified in known myopia genes. a, Distribution of pathogenic (P), likely pathogenic (LP), uncertain significance (VUS), likely benign (LB) and benign (B) variants across all myopia known genes. b, Distribution of PTVs, missense, synonymous variants and other types (including in-frame indels) of all detected variants across genes. c, Distribution of PTVs, missense, synonymous variants and other types of P/LP variants across genes.

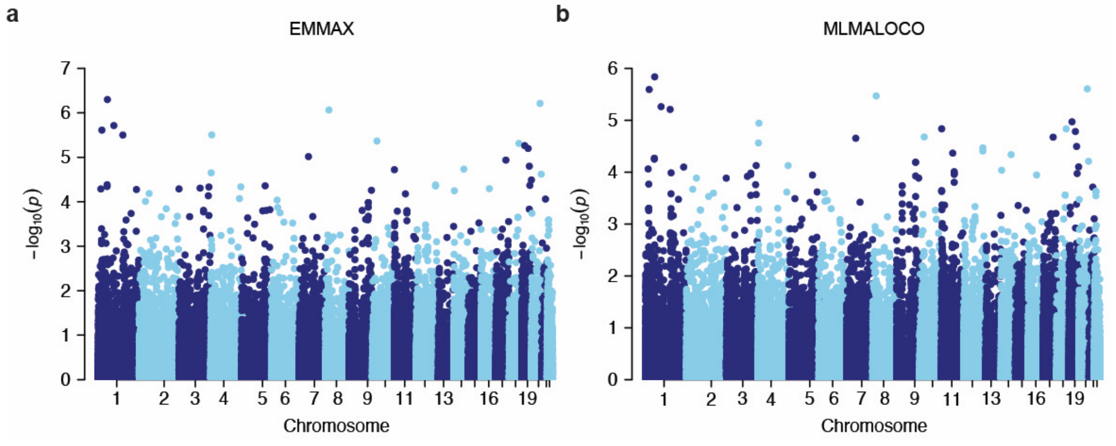

Supplementary Fig. 2. Exome-wide association study for single variants by EMMAX (a) and MLMA-LOCO (b). The threshold of exome-wide correction for multiple testing was set as $P < 4.3 \times 10^{-7}$, which gave all variants in coding regions equal weights and assigned no weight to other variants.

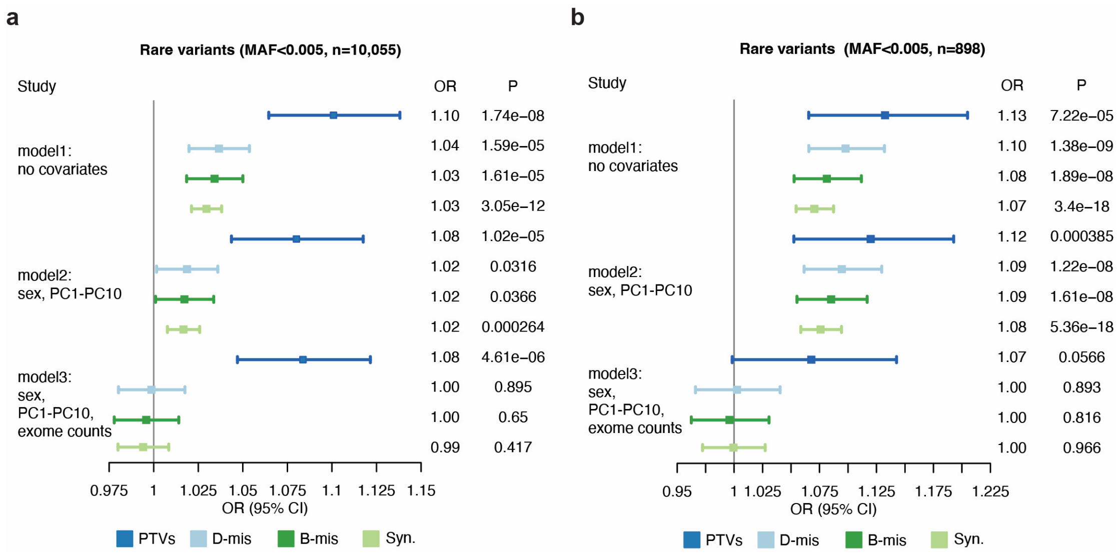

Supplementary Fig. 3. Burden analysis for 449 EM cases and 9606 controls. a, Burden analysis in variants that annotated within different types of protein-coding catalogues for discovery. b, Burden analysis for down-sampling cohort. Significance of association is displayed with the P-values from two-sided Firth logistic regression test; errors bars indicated 95% confidence intervals (CIs) of the corresponding odds ratios. Multiple test correction *P* = 0.01. The graphs display the means and standard deviations.

Model 1: Sample variation. The graph displayed the mean and standard deviation. P-values from firth logistic regression test are also displayed.

Model 2: Sample variation, sample sex, PC1-PC10.

Model 3: Sample variation, sample sex, PC1-PC10, and total exome count (summation of synonymous, benign missense, damaging missense, and PTV).

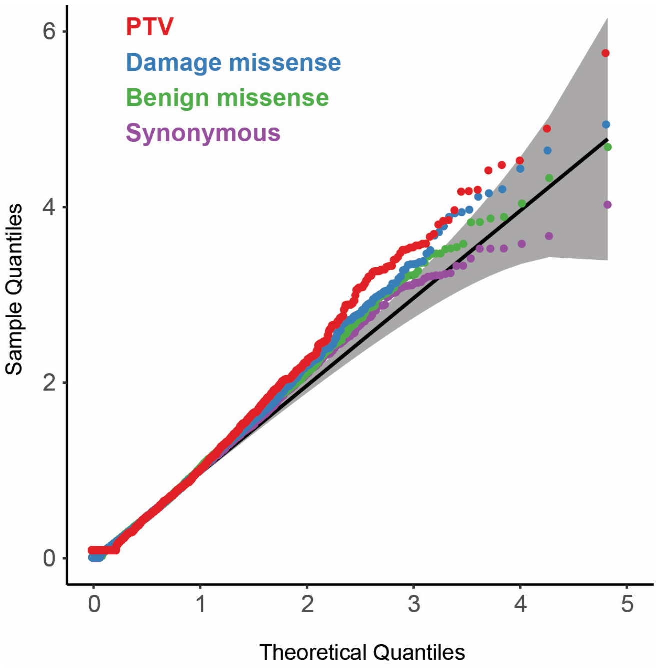

Supplementary Fig. 4. Enrichment of rare variants in 10,587 gene sets derived from Human MSigDB Collections.

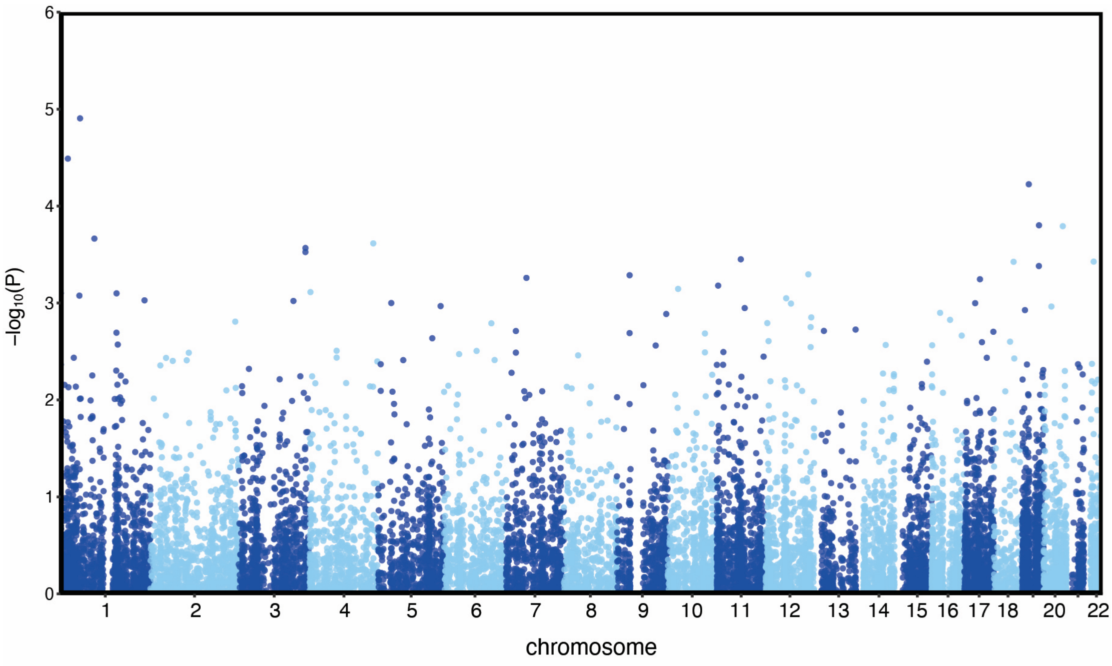

Supplementary Fig. 5. Manhattan plot of MAGMA gene-based genetic association analysis on EM. Bonferroni significance threshold is 0.05/20,000=2.5 × 10^{-6}

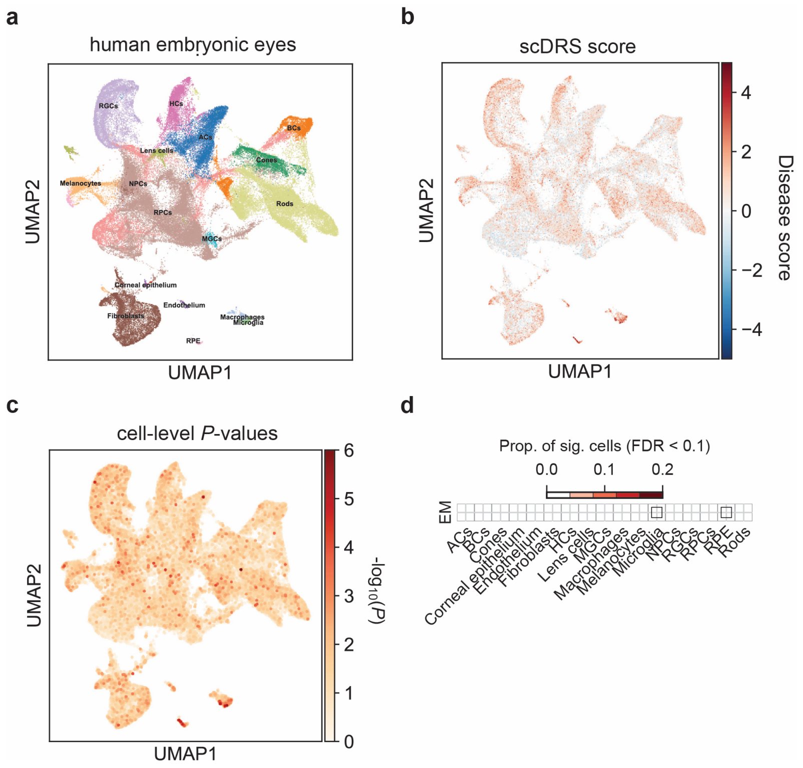

Supplementary Fig. 6. Associations of single-cell of human eyes with EM. (a) Uniform manifold approximation and projection (UMAP) embedding plot shows the cellular component of a scRNA-seq dataset for human embryonic eyes. Abbreviations are as follows: RPCs, retinal progenitor cells; NPCs, neurogenic RPCs; MGCs, Müller glial cells; HCs, horizontal cells; BCs, bipolar cells; ACs, amacrine cells; RGCs, retinal ganglion cells; RPE, retinal pigment epithelia; Endothelium, endothelial cells. (b) Subpopulations of cell-type associated with EM. The color denoted scDRS disease scores. (c) for each cell based on the empirical distribution of the pooled normalized control scores across all control gene sets and all cells. *P*-value for each cell based on the empirical distribution of the pooled normalized control scores across all control gene sets and all cells. The choice of 1,000 for the number of putative disease genes and the choice of 1,000 for the number of control scores are independent. (d) Heatmap colors for each cell type-disease pair denote the proportion of significantly associated cells. *P*-values via one-sided Monte Carlo (MC) test.

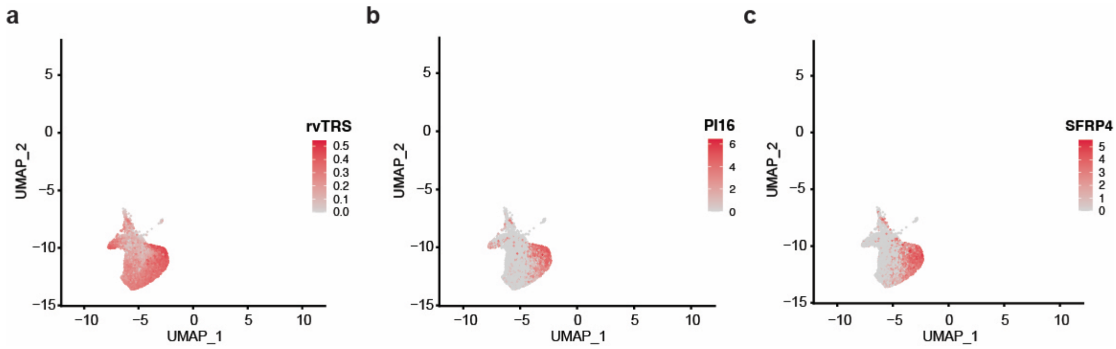

Supplementary Fig. 7. Gene expression of EM-associated fibroblasts. a, UMAP embedding plot shows the per-cell rvTRS. b, UMAP embedding plot shows expression of *PI16.* c, UMAP embedding plot shows *SFRP4* of a scRNA-seq dataset for human embryonic eyes within fibroblasts cluster.

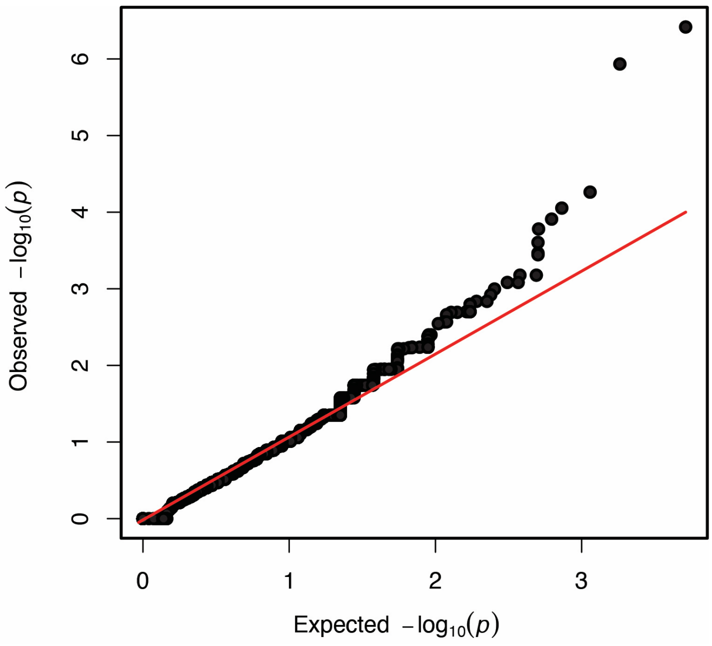

Supplementary Fig. 8. QQ plots for gene-level burden analysis by using two-sided FET.

Two-sided FET for functional variants at MAF < 0.1% in 10,055 Chinese subjects (449 EM and 9606 controls).

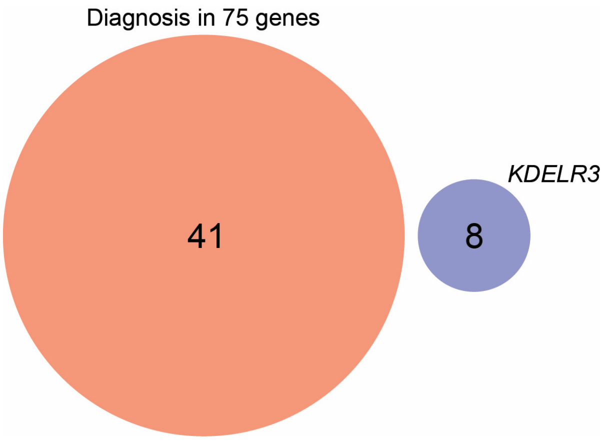

Supplementary Fig. 9. Overlap between cases with *KDELR3* PTVs and ones with diagnostic variants.

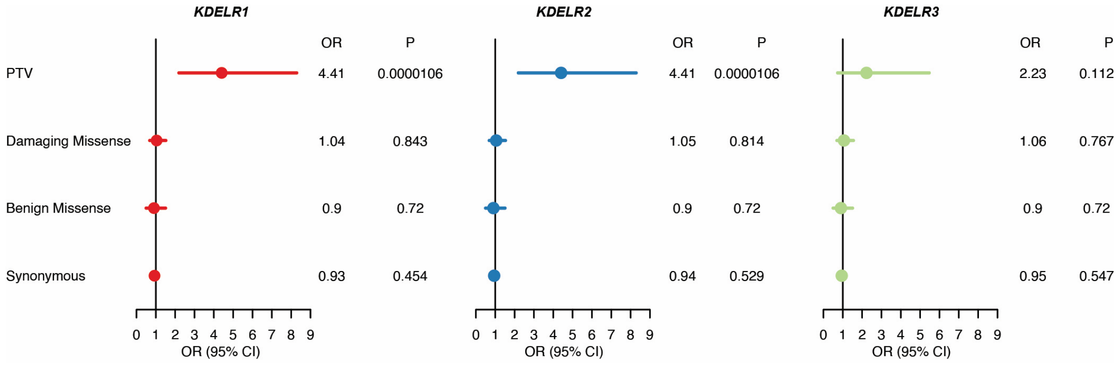

Supplementary Fig. 10. Burden analysis of genes involved in retrograde vesicle-medicated transport, Golgi to ER, when removed *KDELR1*, *KDELR2* or *KDELR3*. PTVs, D-mis, B-mis and synonymous variants within rare variants are displayed. Significance of association is displayed with the P-values from two-sided Firth logistic regression test; errors bars indicated 95% confidence intervals (CIs) of the corresponding odds ratios. The graphs display the means and standard deviations.

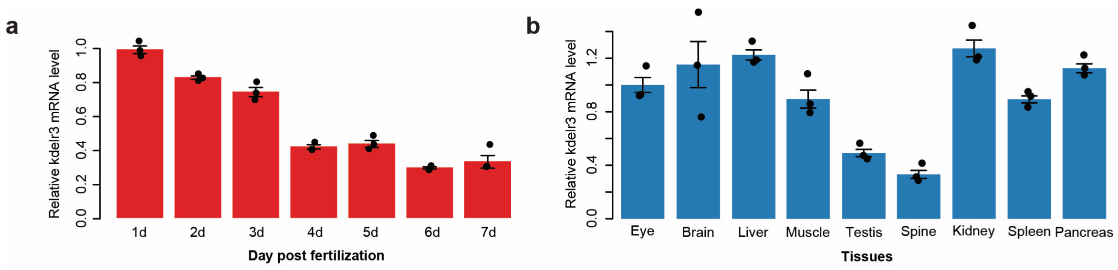

Supplementary Fig. 11. Spatiotemporal expression pattern of kdelr3 in zebrafish. a, The kdelr3 expression decreased from 1 to 7 dpfs. b, Spatial expression pattern of kdelr3 in 3-month-old adult male zebrafish.

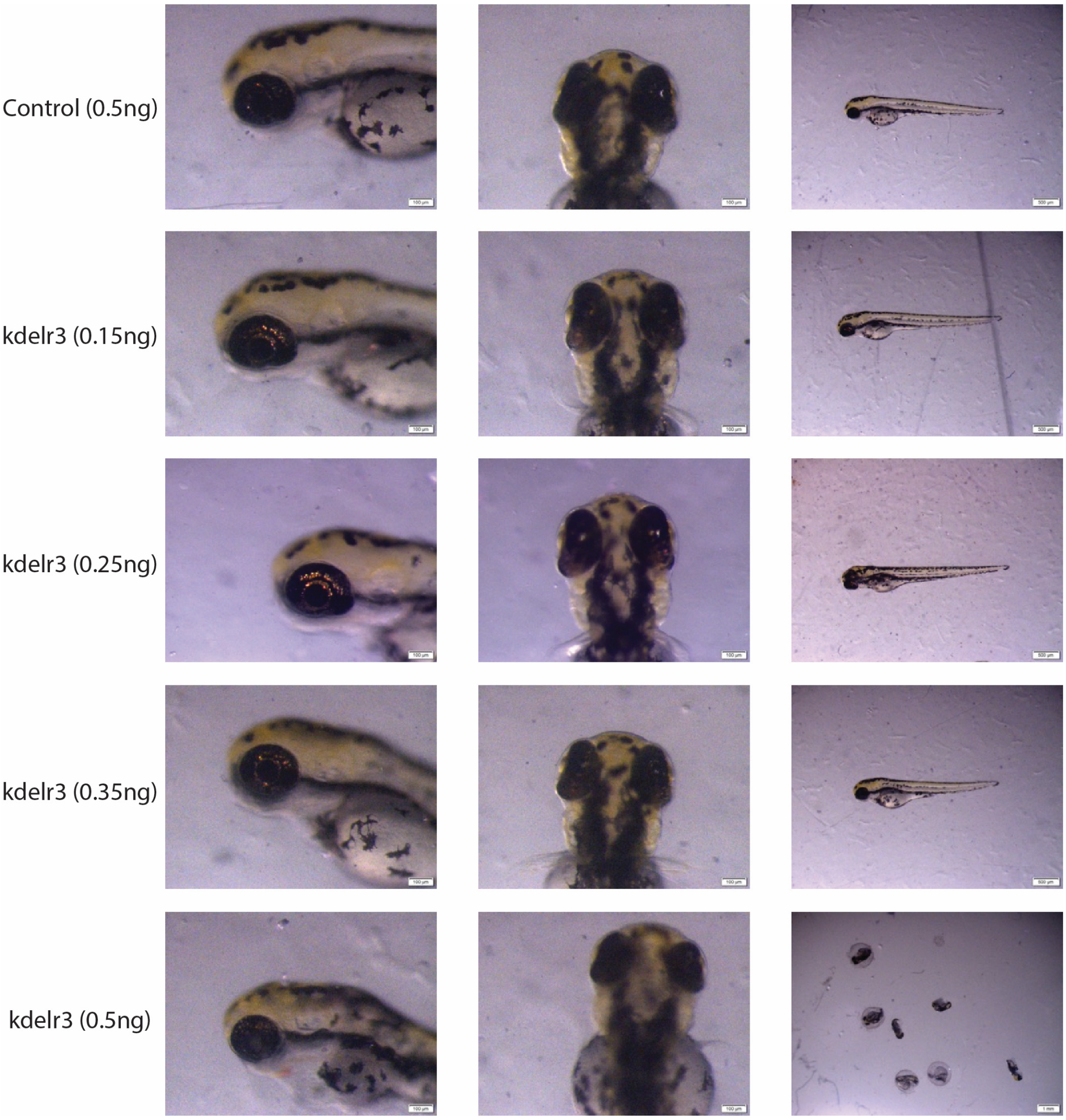

Supplementary Fig. 12. Morphology of kdelr3-deficient zebrafish morphants. Lateral view of zebrafish larvae. Embryos injected with doses of 0.15 to 0.5 ng MOs exhibited apparent microphthalmia.

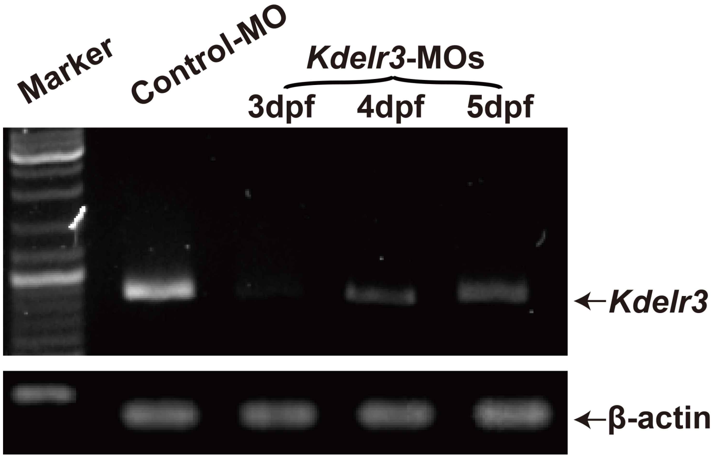

Supplementary Fig. 13. RT-PCR confirmed expression change in *kdelr3*-MO injected zebrafish larvae. 0.5 ng of *kdelr3*-MOs were tested at 3dpf, 4dpf and 5dpf. 0.5 ng of control-MOs were tested at 5dpf.

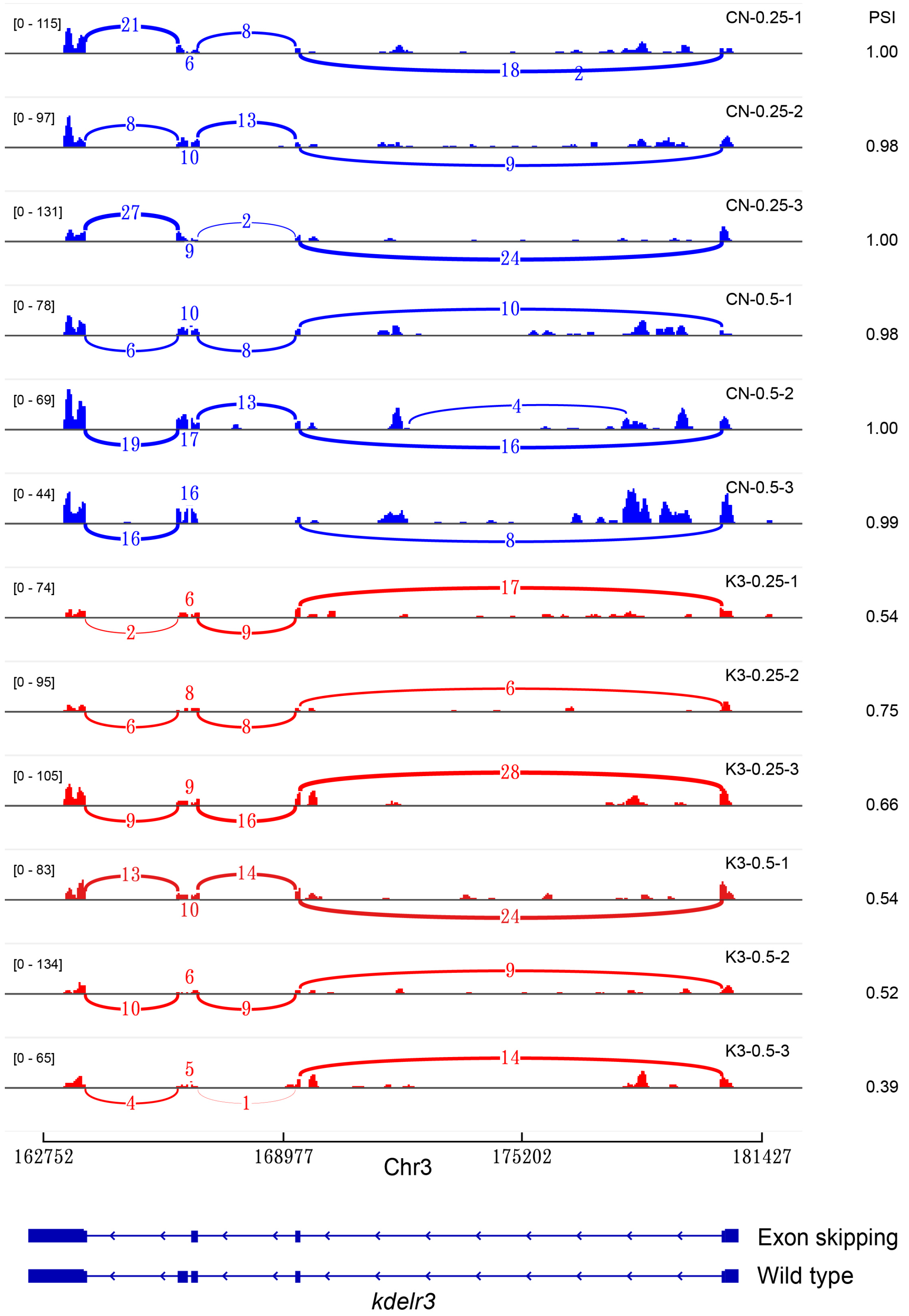

Supplementary Fig. 14. Sashimi plots illustrating target exon (exon 4) in kdelr3-201 (GRCz11) in zebrafish eyeballs treated with *kdelr3* MOs (red sashimi plots) and controls MOs (blue sashimi plots). PSI value is provided for each condition

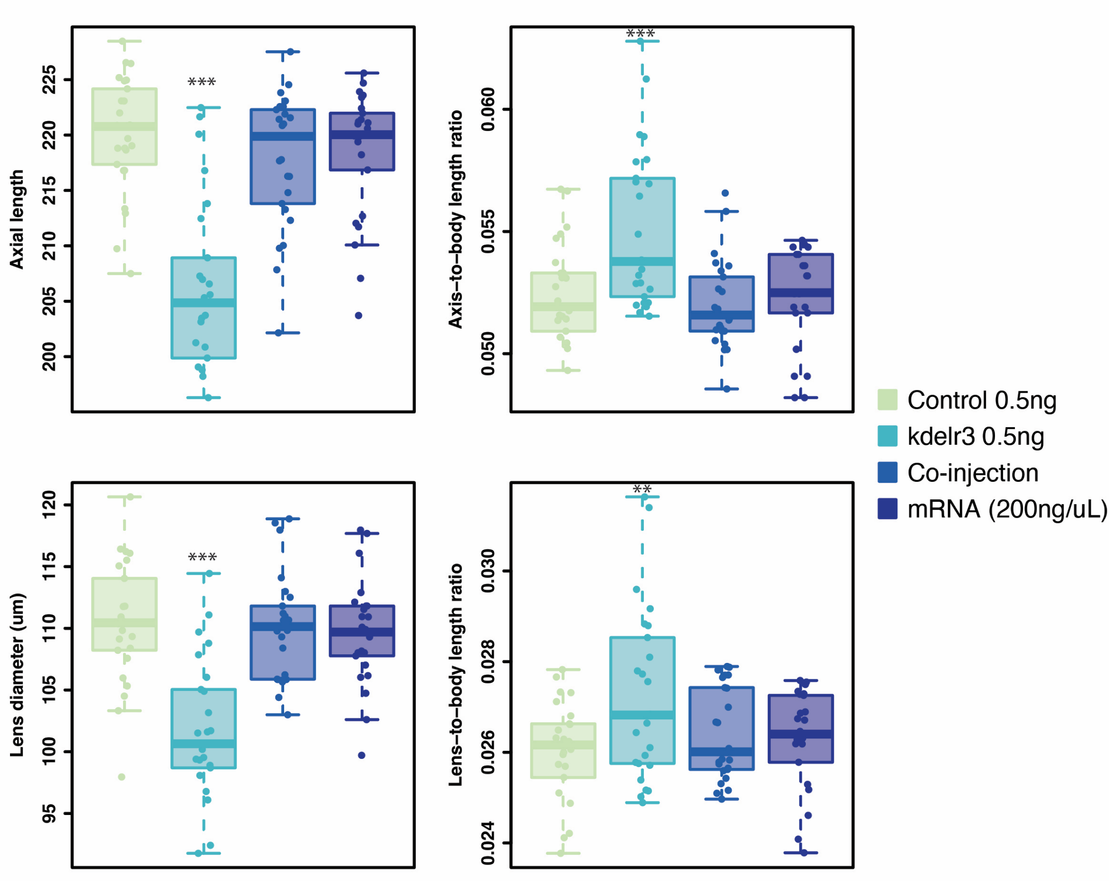

Supplementary Fig. 15. Quantification of eye axis length, the axis-to-body length ratio, lens diameter and lens-to-body length ratio for kdelr3-deficinet and rescue zebrafish. Data was analyzed by two-sided Student's t test, ∗P < 0.05, ∗∗P < 0.01, ∗∗∗P < 0.001 significantly different from control 0.5 ng group (n.s.: non-significant differences). Boxplots display the median, quartiles, and variability of the data.

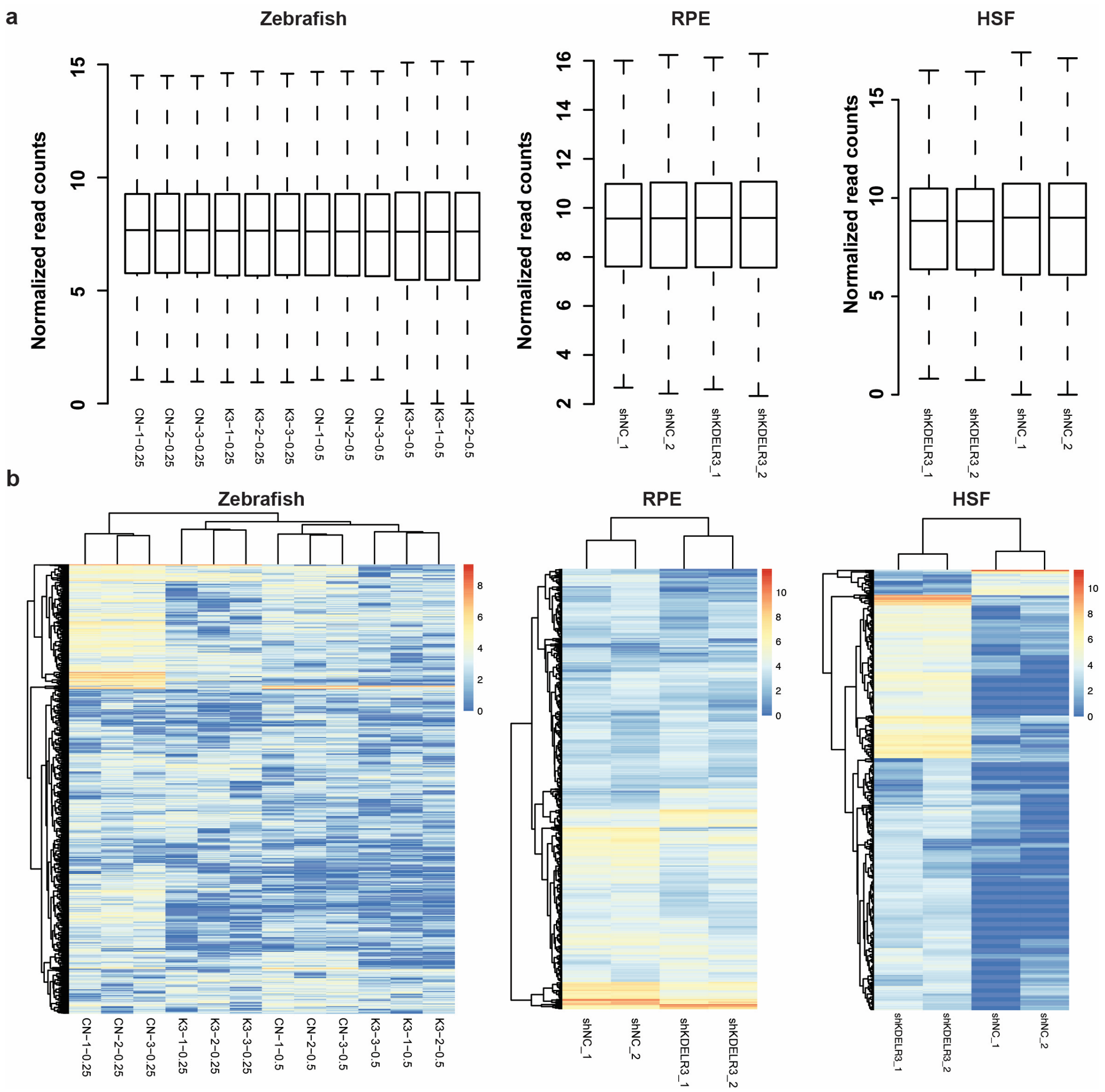

Supplementary Fig. 16. RNA-Seq data quality assessment. a, Box plot comparing the sample level distributions of gene expression data post normalization (Log2 (DESeq count+1)) for zebrafish, RPE cells and HSF cells. **b,** correlation-based clustered Heat-Map depicting relationships across zebrafish, RPE cells and HSF cells, when normalized gene expression data (Log2 (DESeq count +1)) is used for highly variable genes.

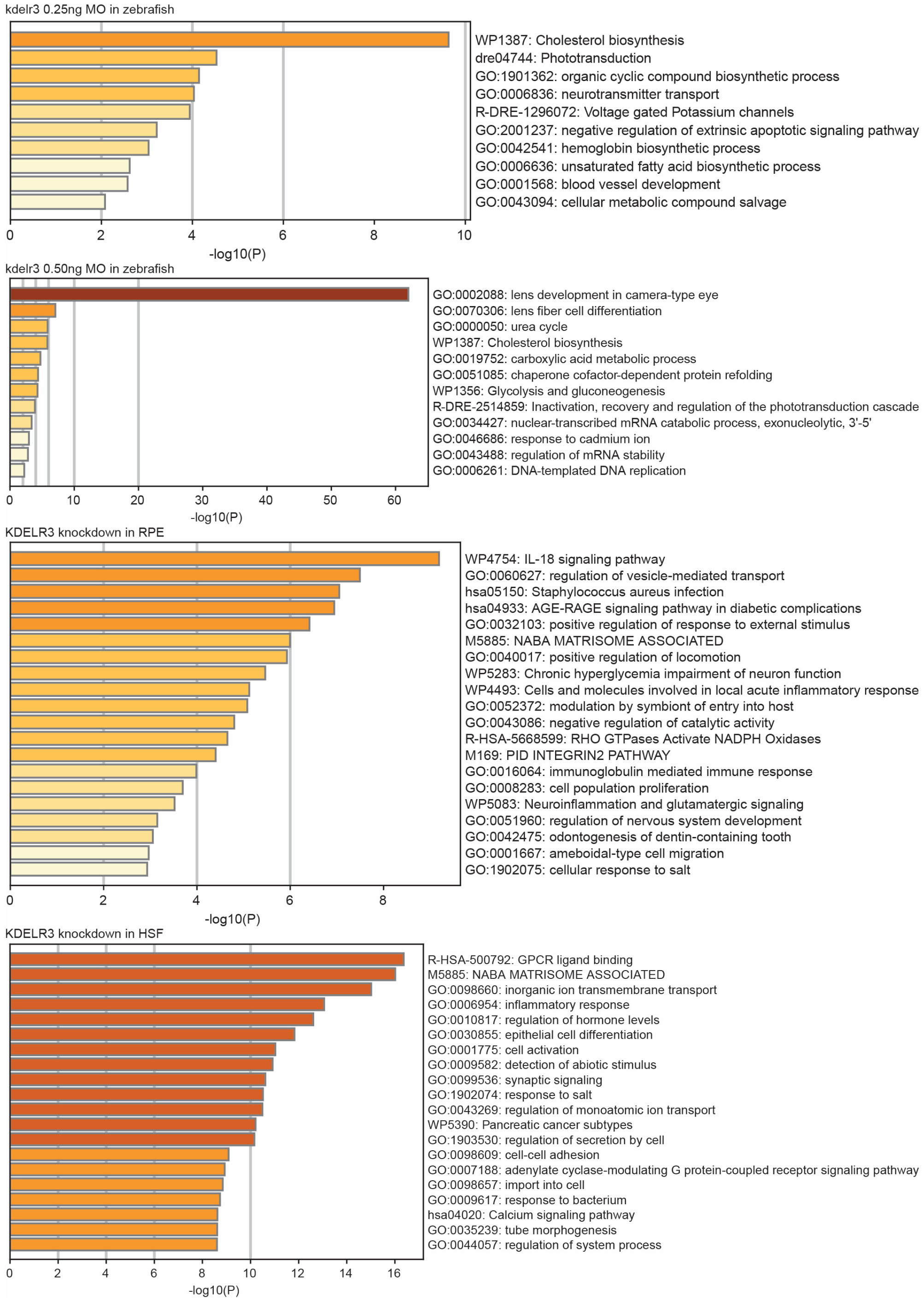

Supplementary Fig. 17. Heat map of Metascape-enriched clusters of upregulated targets in *KDELR3*-deficient transcriptome. Each cluster contains multiple gene sets to eliminate redundancy. Analysis used genes meeting an FDR q < 0.05 threshold. The p-value is defined as the probability of obtaining *n* or more pathway members, forming a cumulative hypergeometric distribution.

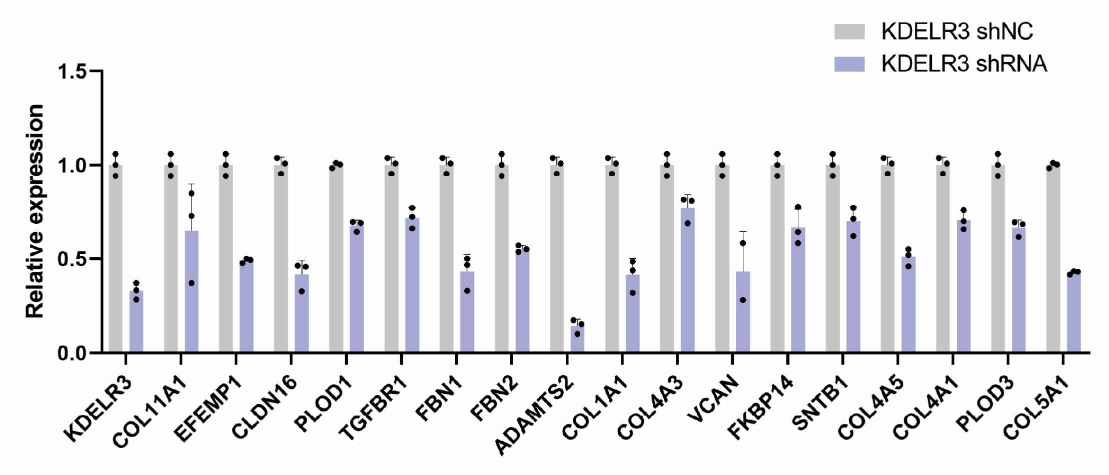

Supplementary Fig. 18. qRT-PCR validation analysis. Barplot shows the mRNA expression fold change of ECM organization-associated genes in the control vs *KDELR3*-deficient RPE cells. Each group has three biological replications. Data represent mean±s.d. and *P*-value was generated by using two-sided Student's t test. *P < 0.05; **P < 0.01; ***P < 0.001.

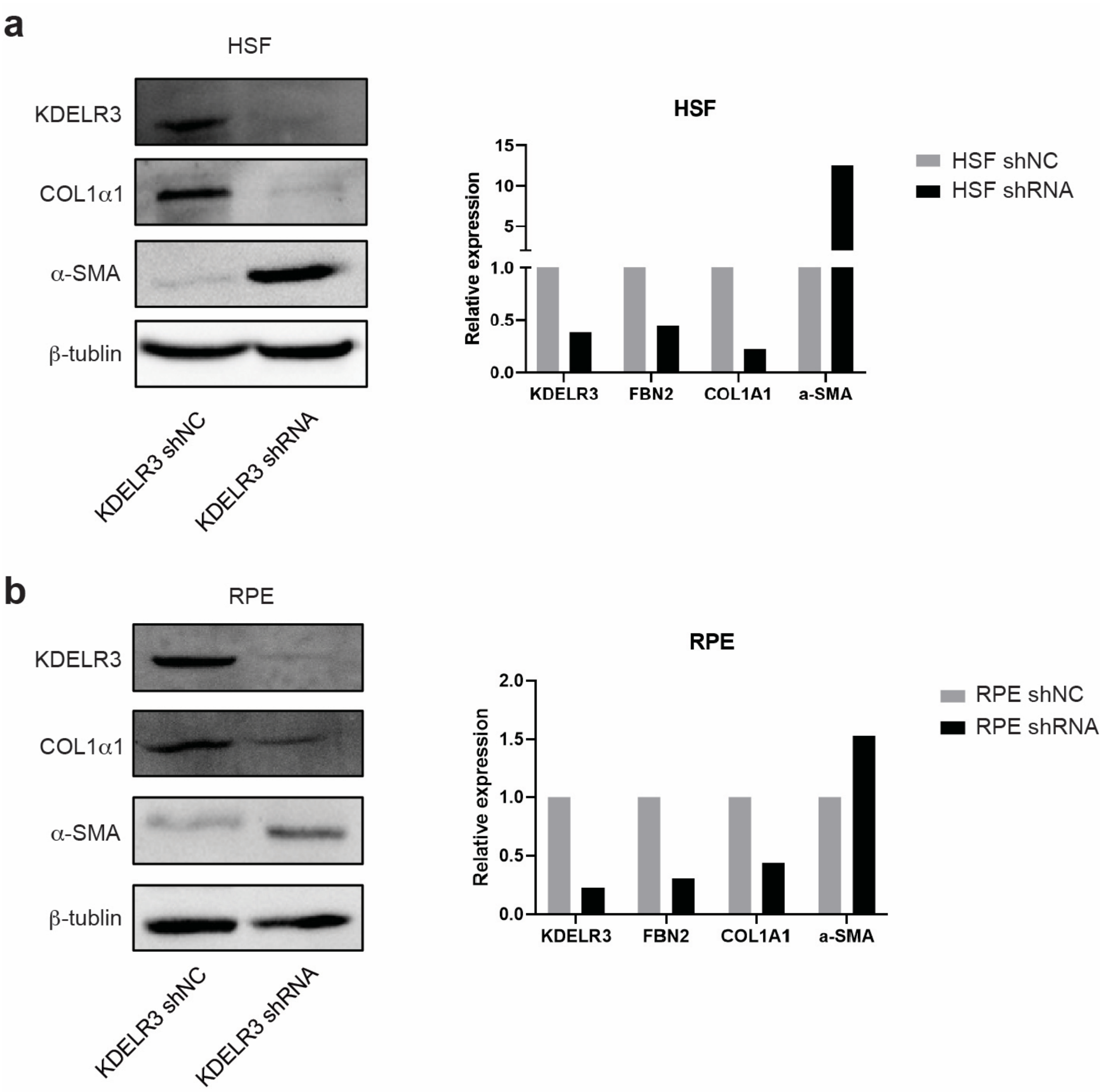

Supplementary Fig. 19. Western blot analysis. **a,** Protein levels of scleral KDELR3, COL1α1, and α-SMA were detected by Western blot in *KDELR3*-deficient HSF cells. b, Protein levels of scleral KDELR3, COL1α1, and α-SMA in RPE cells.

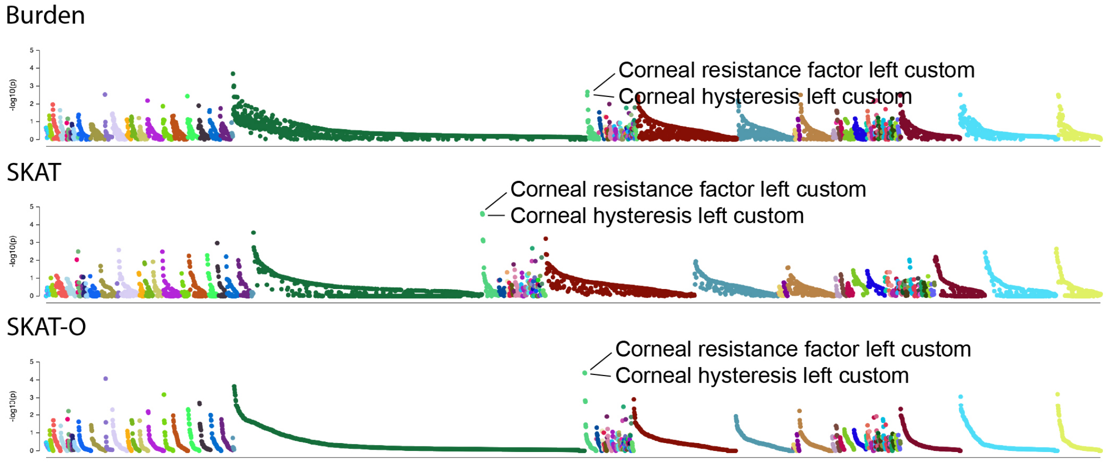

Supplementary Fig. 20. PheWAS Manhattan Plot. Each data point represents phenotypic associations with *KDELR3* in pLoF model. The data points are grouped and color-coded by phenotype groups (x-axis) and -log10(p-value) (y-axis).

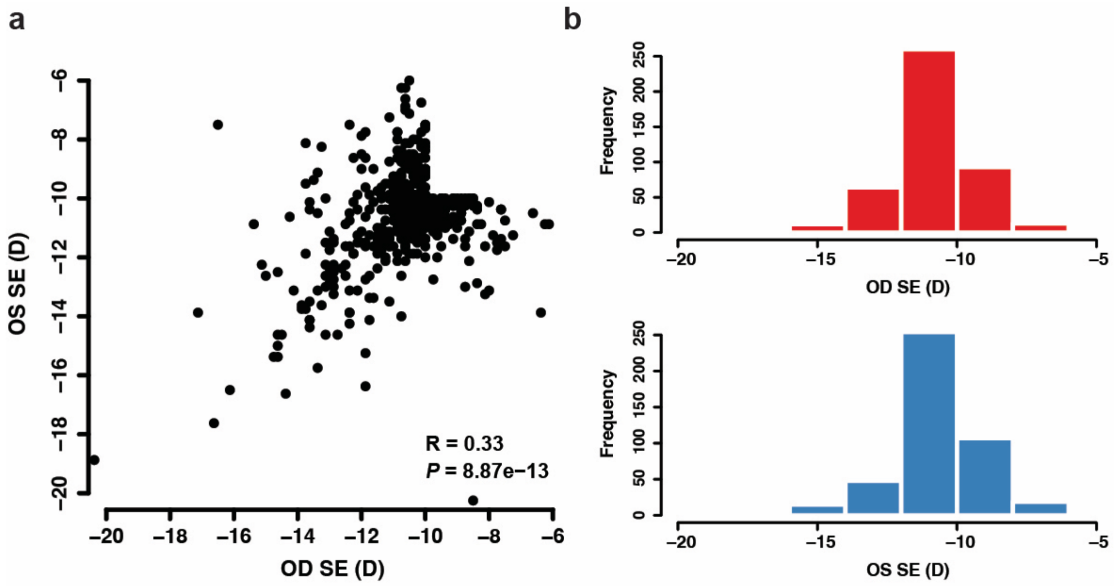

Supplementary Fig. 21. Distribution of spherical equivalent refraction of 449 EM. a, Correlation of spherical equivalent refractive error between right and left eye in all recruited cases. b, Distribution of spherical equivalent refractive error in both of eyes for 449 EM cases as assessed by visual acuity and autorefraction testing. Negative values indicate myopia.

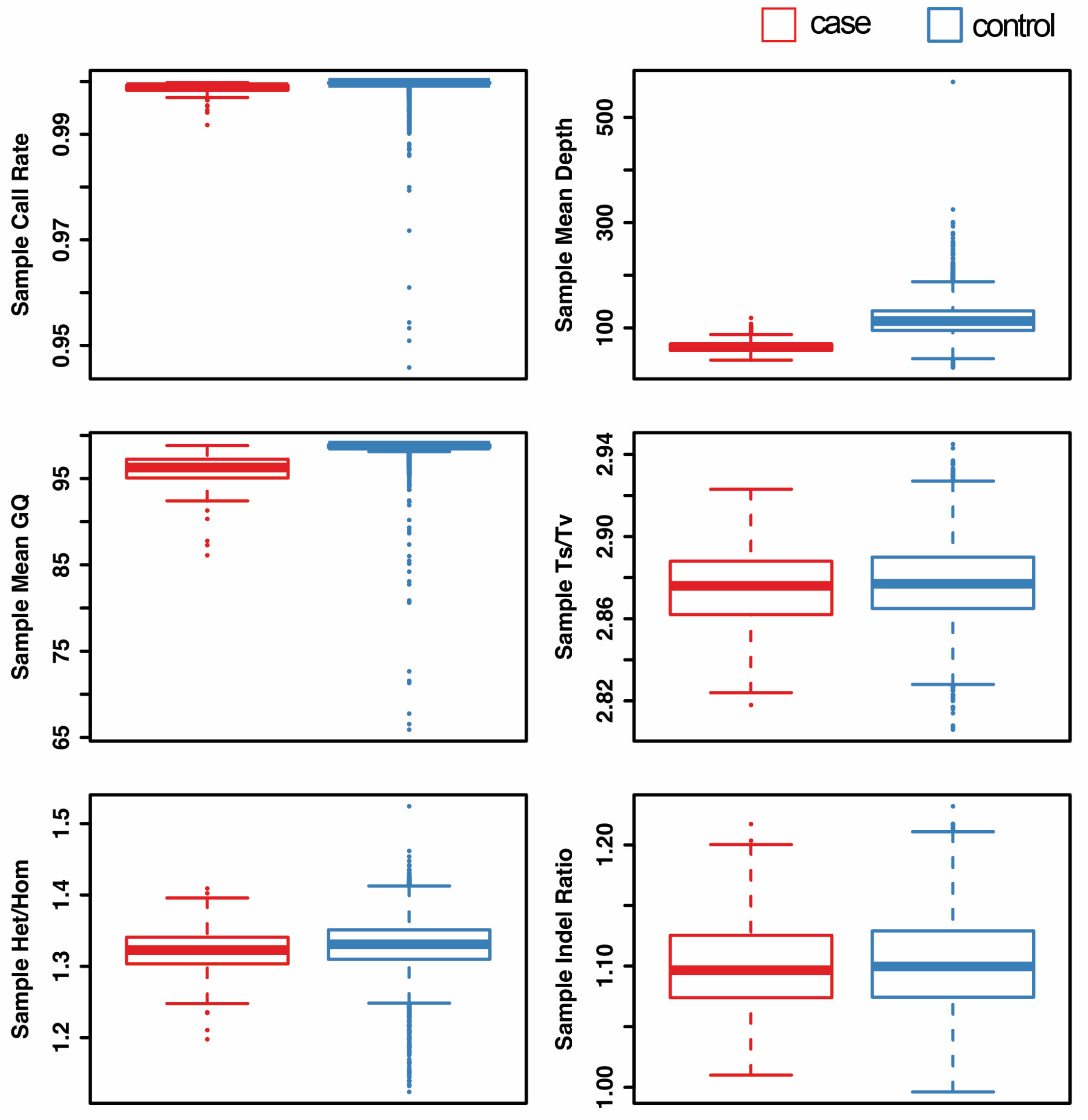

Supplementary Fig. 22. Initial sample quality control analysis. Distribution of sample call rate, sample mean depth, sample mean genotype quality, sample transition to transversion ratio, sample heterozygous to homozygous ratio and sample insertion to deletion ratio. The box and whisker plots display the mean, minimum, and maximum.

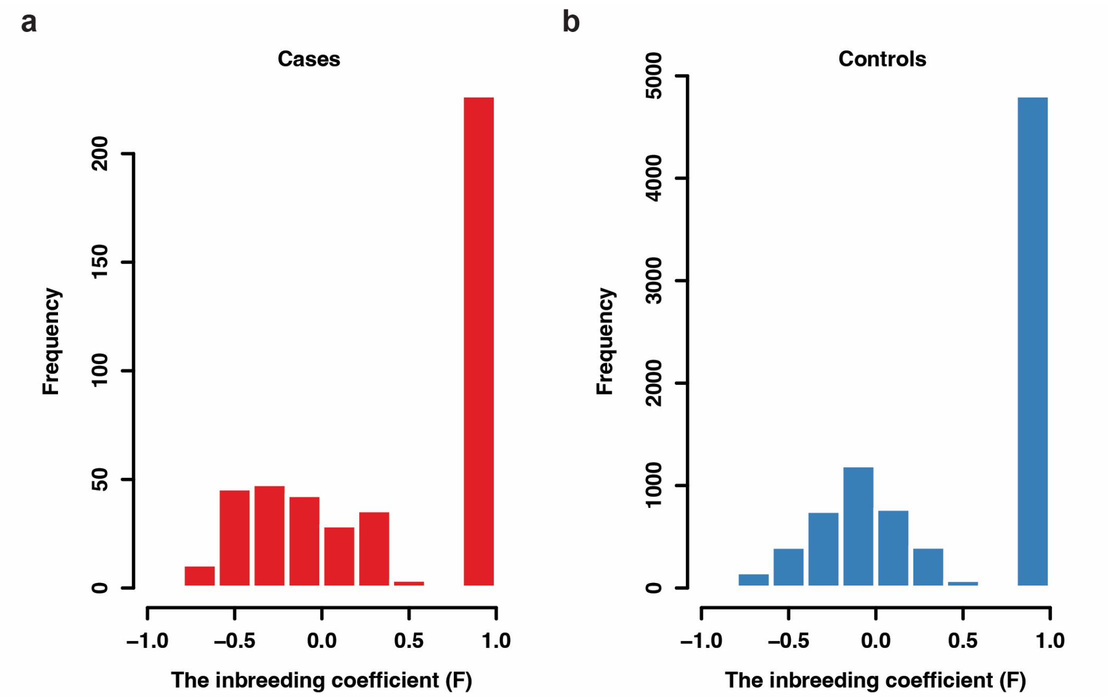

Supplementary Fig. 23. Distribution of the inbreeding coefficient for 449 EM cases (a) and 9606 controls (b). Samples with an X chromosome inbreeding coefficient > 0.8 were classified as males, while samples with an X chromosome inbreeding coefficient < 0.4 were classified as females. Samples with an X chromosome inbreeding coefficient between < 0.8 and > 0.4 which classified as ambiguous sex status, were excluded from the dataset.

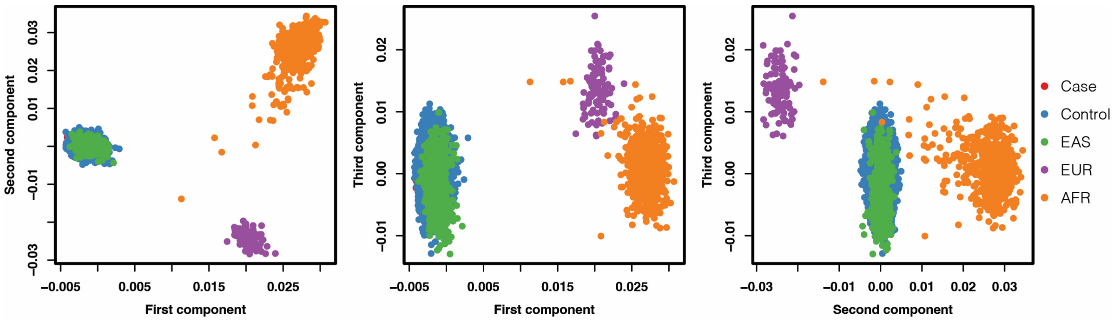

Supplementary Fig. 24. Principal component analysis with 1000 Genomes. PCA was run on the study samples along with 1000 Genomes (1KG) phase 3 super populations to infer genetic ancestry. Genetic ancestry of the EM cases and controls were predicted by the Random Forest classifier based on top 6 PCs, using 1KG samples as the training data. Individuals were assigned to a particular 1KG-ancestry with a predicted probability >0.9, as depicted in the figure.

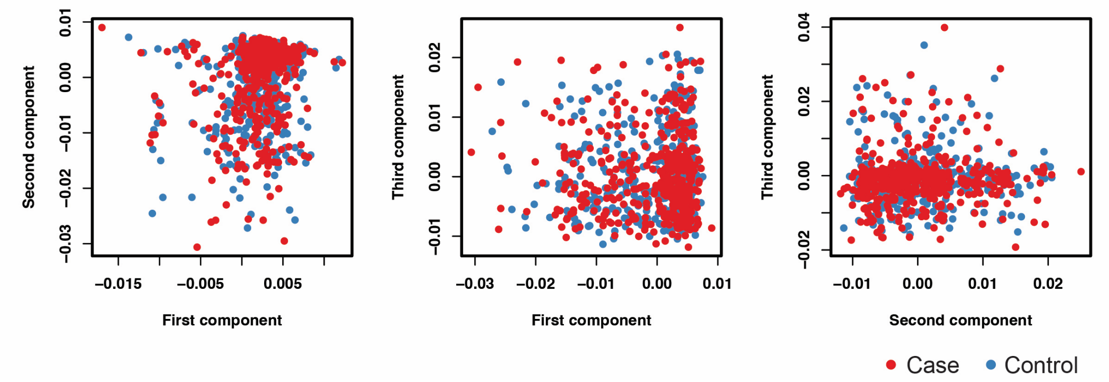

Supplementary Fig. 25. PCA on 449 EM cases and 9606 controls samples. PCA after down-sampling by removed controls not pair-matched with cases based on top 3 PCs.
