# Supplementary material for: Exome-wide association study identifies KDELR3 mutations in extreme myopia
Source: Nat Commun. 2024 Aug 7;15:6703. doi: 10.1038/s41467-024-50580-x (PMC11306401; doi:10.1038/s41467-024-50580-x)
Supplement: Supplementary file 3 — Description of Additional Supplementary Files [file 41467_2024_50580_MOESM3_ESM.pdf]

Description of Additional Supplementary Files

File Name: Supplementary Data 1

Description: Sample QC and number of sample dropout at each QC step.

File Name: Supplementary Data 2

Description: Variants identified from exome sequencing

File Name: Supplementary Data 3

Description: Genes related with HM According to the OMIM.

File Name: Supplementary Data 4

Description: Pathogenic and likely pathogenic variant in known HM genes.

File Name: Supplementary Data 5

Description: Enriched terms of known HM genes. The P-value is defined as the probability of obtaining n or more pathway members, forming a cumulative hypergeometric distribution.

File Name: Supplementary Data 6

Description: Grouping of functional consequences of the called sites.

File Name: Supplementary Data 7

Description: Gene-set enrichment analysis for rare PTVs performed by two-tailed FET.

File Name: Supplementary Data 8

Description: Gene-set enrichment analysis for rare D-mis performed by two-tailed FET.

File Name: Supplementary Data 9

Description: Gene-set enrichment analysis for rare benign missense performed by two-tailed FET.

File Name: Supplementary Data 10

Description: Gene-set enrichment analysis for rare synonymous performed by two-tailed FET.

File Name: Supplementary Data 11

Description: Top 10% most specific genes per cell type for the human embryonic eye.

File Name: Supplementary Data 12

Description: Polygenic burden test for top 10% markers in each cell types by Firth logistic regression test.

File Name: Supplementary Data 13

Description: Mark genes seleceted by comparing EM-associated cells (rvTRS > 0.4) vs non-EM-associated cells (rvTRS < 0.1) based on the non-parametric Wilcoxon rank sum test.

File Name: Supplementary Data 14

Description: Gene-based rare variant association study performed by two-tail FET, burden test and SKAT.

File Name: Supplementary Data 15

Description: Summary of enrichment analysis in DisGeNET.

File Name: Supplementary Data 16

Description: Enriched terms of top100 EM candidate genes. *P*-values are calculated based on the accumulative hypergeometric distribution.

File Name: Supplementary Data 17

Description: Percent spliced in (Psi) values for differentially skipped exon in kdelr3-MO zebrafish eyeballs versus control zebrafish eyeballs.

File Name: Supplementary Data 18

Description: Significant DEGs following 0.25ng KDELR3 deficiency in zebrafish based on DESeq2.

File Name: Supplementary Data 19

Description: Significant DEGs following 0.5ng KDELR3 deficiency in zebrafish based on DESeq2.

File Name: Supplementary Data 20

Description: Significant DEGs following KDELR3 deficiency in RPE based on DESeq2.

File Name: Supplementary Data 21

Description: Significant DEGs following KDELR3 deficiency in HSF based on DESeq2.

File Name: Supplementary Data 22

Description: List of the GO terms following 0.25ng KDELR3 deficiency in zebrafish. *P*-values are calculated based on the accumulative hypergeometric distribution.

File Name: Supplementary Data 23

Description: List of the GO terms following 0.5ng KDELR3 deficiency in zebrafish. *P*-values are calculated based on the accumulative hypergeometric distribution.

File Name: Supplementary Data 24

Description: List of the GO terms following KDELR3 deficiency in RPE. *P*-values are calculated based on the accumulative hypergeometric distribution.

File Name: Supplementary Data 25

Description: List of the GO terms following KDELR3 deficiency in HSF. *P*-values are calculated based on the accumulative hypergeometric distribution.

File Name: Supplementary Data 26

Description: qPCR primer sequences for zebrafish.

File Name: Supplementary Data 27

Description: Primer sequences for real-time PCR.

File Name: Supplementary Data 28

Description: Primer information for shRNA and qPCR.
