# Supplementary material for: Exome-wide association study identifies KDELR3 mutations in extreme myopia
Source: Nat Commun. 2024 Aug 7;15:6703. doi: 10.1038/s41467-024-50580-x (PMC11306401; doi:10.1038/s41467-024-50580-x)
Supplement: Supplementary file 5 — Reporting Summary [file 41467_2024_50580_MOESM5_ESM.pdf]

nature portfolio

Corresponding author(s): Xiaoguang Yu, Jia Qu, Jianzhong Su

Last updated by author(s): Apr 24, 2024

Reporting Summary

Nature Portfolio wishes to improve the reproducibility of the work that we publish. This form provides structure for consistency and transparency in reporting. For further information on Nature Portfolio policies, see our Editorial Policies and the Editorial Policy Checklist.

Statistics

For all statistical analyses, confirm that the following items are present in the figure legend, table legend, main text, or Methods section.

n/a	Confirmed	
☐	☒	The exact sample size (*n*) for each experimental group/condition, given as a discrete number and unit of measurement
☐	☒	A statement on whether measurements were taken from distinct samples or whether the same sample was measured repeatedly
☐	☒	The statistical test(s) used AND whether they are one- or two-sided<br>*Only common tests should be described solely by name; describe more complex techniques in the Methods section.*
☐	☒	A description of all covariates tested
☐	☒	A description of any assumptions or corrections, such as tests of normality and adjustment for multiple comparisons
☐	☒	A full description of the statistical parameters including central tendency (e.g. means) or other basic estimates (e.g. regression coefficient) AND variation (e.g. standard deviation) or associated estimates of uncertainty (e.g. confidence intervals)
☐	☒	For null hypothesis testing, the test statistic (e.g. *F*, *t*, *r*) with confidence intervals, effect sizes, degrees of freedom and *P* value noted<br>*Give P values as exact values whenever suitable.*
☒	☐	For Bayesian analysis, information on the choice of priors and Markov chain Monte Carlo settings
☒	☐	For hierarchical and complex designs, identification of the appropriate level for tests and full reporting of outcomes
☐	☒	Estimates of effect sizes (e.g. Cohen's *d*, Pearson's *r*), indicating how they were calculated

Our web collection on statistics for biologists contains articles on many of the points above.

Software and code

Policy information about availability of computer code

Data collection	No software was used for data collection.
Data analysis	The FastQC package was used to assess the quality-score distribution of the sequencing reads. Read sequences were mapped to human genome build 37 (GRCh37)51, 52 using the Burrows-Wheeler Aligner (BWA 0.7.12). And Sambamba 0.6.6 (https://lomereiter.github.io/sambamba/) was used for sorting by chromosome coordinates and marking duplicates. After alignment by BWA, the reads were subjected to recalibration using the Genome Analysis Toolkit (GATK v. 4.0.11.0). Haplotype calling was performed by using HaplotypeCaller in GATK v.4.0 in GVCF mode according to the best practice. GVCF were merged and joint genotyped with GenotypeGVCFs in GATK v.4.0 to produce a combined VCF file for further analysis. This pipeline detected SNVs and small insertion or deletion (indel) variants from exome sequence data. We applied standard variant-level and individual-level quality controls. Variant calling accuracy was estimated using the GATK Variant Quality Score Recalibration (VQSR) approach. Then, we excluded variants for further analysis if (1) they were located inside of low-complexity regions; (2) they failed in GATK VQSR metric; (4) they had calling rates < 90%; (5) Hardy-Weinberg Equilibrium (HWE) test P-value < 10-6 on the basis of the combined case and control cohort; (6) Genotypes with a genotype depth (DP) < 10 and genotype quality (GQ) < 20; and (7) heterozygous genotype calls with an allele balance > 0.8 or < 0.2. After that, we excluded samples with a low average call rate (< 0.9), low mean sequencing depth (< 10), or low mean genotype quality (< 65). Outliers (> 4 standard deviation [SD] from the mean) of the transition/transversion ratio, heterozygous/homozygous ratio, or insertion/deletion ratio within each cohort were further discarded. Samples with an X chromosome inbreeding coefficient > 0.8 were classified as males, while samples with an X chromosome inbreeding coefficient < 0.4 were classified as females. Samples between <0.8 and >0.4 which classified as ambiguous sex status, were excluded from the dataset. We detected population outliers and stratification using a method based on principal component analysis (PCAs) with a subset of high-confidence single-nucleotide polymorphisms (MAF>1%) in the exome capture region. Only retained individuals of East Asian (EAS) ancestry were retained, which were classified by a random forest algorithm with 1000 Genomes data. Within the EAS population, we down-sampling cohorts (449 cases and 449 controls) by removed the controls that were not well matched with cases on the basis of the top three PCs by PCAmatchR. We included only

unrelated individuals (identity by descent proportion < 0.2) using PLINK 1.07. After QC, we retained 449 cases and 9,606 controls.
The source code in this study was provided in an open-source repository in Github (https://github.com/sulab-wmu/MAGIC-PIPELINE and https://github.com/sulab-wmu/MAGIC).

For manuscripts utilizing custom algorithms or software that are central to the research but not yet described in published literature, software must be made available to editors and reviewers. We strongly encourage code deposition in a community repository (e.g. GitHub). See the Nature Portfolio guidelines for submitting code & software for further information.

Data

Policy information about availability of data

All manuscripts must include a data availability statement. This statement should provide the following information, where applicable:

- Accession codes, unique identifiers, or web links for publicly available datasets
- A description of any restrictions on data availability
- For clinical datasets or third party data, please ensure that the statement adheres to our policy

Individual-level data are not publicly available due to ethical and legal restrictions related to the Wenzhou Medical University. VCF files have been deposited to Genome Variation Map (http://bigd.big.ac.cn/gsa; GVM000296) and RNA-seq fastq files have been deposited to Genome Sequence Archive (http://bigd.big.ac.cn/gsa; PRJCA020266) in BIG Data Center, Beijing Institute of Genomics (BIG), Chinese Academy of Sciences and are publicly available as of the data of publication. The datasets of genotype information are available from the corresponding author on request.

Research involving human participants, their data, or biological material

Policy information about studies with human participants or human data. See also policy information about sex, gender (identity/presentation), and sexual orientation and race, ethnicity and racism.

Reporting on sex and gender	Our study included 449 individuals with Extreme myopia (EM) at the Eye Hospital of Wenzhou Medical University (Zhejiang Eye Hospital, Wenzhou, China). The number of male and female patients is 226 and 223, respectively.
Reporting on race, ethnicity, or other socially relevant groupings	All the participants involved in this study were of the Chinese population. This study did not analyze race, ethnicity, or other socially relevant categorization variables. We detected population outliers and stratification using a method based on principal component analysis (PCAs) with a subset of high-confidence single-nucleotide polymorphisms (MAF>1%) in the exome capture region. Only retained individuals of East Asian (EAS) ancestry were retained, which were classified by a random forest algorithm with 1000 Genomes data
Population characteristics	Population characteristics have been summarized in Supplementary Table1.
Recruitment	The Myopia Associated Genetics and Intervention Consortium (MAGIC) is a large-scale genomic consortium integrating myopia cohorts and sequencing data from many investigators. Over the past several years, MAGIC has been able to collected samples at the Eye Hospital of Wenzhou Medical University (Zhejiang Eye Hospital) through the Institute of Biomedical Big Data5. We recruited approximately ten thousand Chinese schoolchildren with high myopia aged from 6 to 18 from MAGIC. EM was defined as an uncorrected visual acuity of 20/25 or less and a spherical equivalent refraction (SER) of -10.0 D or less. Individuals with EM were basically diagnosed by visual acuity and autorefraction testing
Ethics oversight	Research performed on samples and data of human origin was conducted according to protocols approved by the institutional review boards of the Eye Hospital of Wenzhou Medical University, and informed consent was obtained from all subjects.

Note that full information on the approval of the study protocol must also be provided in the manuscript.

Field-specific reporting

Please select the one below that is the best fit for your research. If you are not sure, read the appropriate sections before making your selection.

☒ Life sciences ☐ Behavioural & social sciences ☐ Ecological, evolutionary & environmental sciences

For a reference copy of the document with all sections, see nature.com/documents/nr-reporting-summary-flat.pdf

Life sciences study design

All studies must disclose on these points even when the disclosure is negative.

Sample size	As there is no pre-specified sample size for the discovery cohort was determined, we consecutively enrolled a total of 467 EM and 11,375 controls from the Myopia Associated Genetics and Intervention Consortium (MAGIC) project.
Data exclusions	After stringent quality control, we used WES data from 449 EM-affected individuals and 9606 health control subjects in the initial discovery stage. Data exclusions have been summarized in Supplementary Table1.
Replication	Idependent validation in the other subjects of MAGIC.

Randomization	This study was not randomized because this is an observational case-control study without intervention. To avoid selection bias, the patients with EM and gender- and age-matched healthy controls were consecutively enrolled.
Blinding	Not applicable to this study, as this study is not an interventional study.

Reporting for specific materials, systems and methods

We require information from authors about some types of materials, experimental systems and methods used in many studies. Here, indicate whether each material, system or method listed is relevant to your study. If you are not sure if a list item applies to your research, read the appropriate section before selecting a response.

Materials & experimental systems

n/a	Involved in the study
☐	☒ Antibodies
☐	☒ Eukaryotic cell lines
☒	☐ Palaeontology and archaeology
☐	☒ Animals and other organisms
☒	☐ Clinical data
☒	☐ Dual use research of concern
☒	☐ Plants

Methods

n/a	Involved in the study
☒	☐ ChIP-seq
☒	☐ Flow cytometry
☒	☐ MRI-based neuroimaging

Antibodies

Antibodies used	Recoverin (RCVRN) antibody (rabbit; 1:1000; proteintech), anti-mouse IgG secondary antibodies conjugated with Alexa Fluor 594 (1:200), anti-KDELR3 (proteintech, 27632-1-AP, 1:500), anti-COL1A1 (proteintech, 67288-1-Ig,1:5000), anti-a-SMA (Abcam, ab5694, 1:500), anti-β-tublin (proteintech, 10094-1-AP, 1:5000).
Validation	optimization of the antibodies was carried out on human cell lines RPE and human scleral fibroblasts, with assessment by an experienced consultant biologists.

Eukaryotic cell lines

Policy information about cell lines and Sex and Gender in Research

Cell line source(s)	The human cell lines RPE and human scleral fibroblasts (HSF) were provided by Dr. Yutaka Shimada (Kyoto University, Kyoto, Japan) and Dr. Xiangtian Zhou (Wenzhou Medical University,Wenzhou, China), respectively.
Authentication	n/a
Mycoplasma contamination	n/a
Commonly misidentified lines (See ICLAC register)	n/a

Animals and other research organisms

Policy information about studies involving animals; ARRIVE guidelines recommended for reporting animal research, and Sex and Gender in Research

Laboratory animals	Husbandry adult zebrafish of the Tg(kdrl:mCherry), Tg(gad1b:mCherry) and Tg(gfap-eGFP) strain were obtained from the China Zebrafish Resource Center (CZRC Catalog ID CZ921).
Wild animals	n/a
Reporting on sex	n/a
Field-collected samples	n/a
Ethics oversight	All experiments were carried out in accordance with the Association for Research on Vision and Ophthalmology's statement on the Use of Animals in Ophthalmic and Vision Research and were approved by the Institutional Animal Care and Use Committee of Wenzhou Medical University.

Note that full information on the approval of the study protocol must also be provided in the manuscript.

Plants

Seed stocks	n/a
Novel plant genotypes	n/a
Authentication	n/a
